# How to deal with runaway metastatic disease?

**DOI:** 10.18632/oncotarget.28609

**Published:** 2024-07-12

**Authors:** Justine Paris, Guilhem Bousquet

**Keywords:** metastatic disease, PROM2, biomarker, tumor growth models

Metastatic disease remains a major issue in daily practice in oncology because the outcome is still often fatal. In the last twenty years, the concept of chemo- and immuno-curability with long survivors has become a reality, even at advanced stages of the disease [1]. Despite this, most patients develop resistance to a first-line treatment, with frequent runaway metastatic disease leading to ever shorter durations of treatment response, and finally to death.

There are several mathematical models for tumor growth (Gompertz, von Bertalanffy, exponential, logistic etc.), mainly for primary tumors, but with limited data to model the dynamics of metastatic growth in cancer patients [2]. In the sigmoidal growth curves as proposed by the classic Gompertz model, tumor growth is initially exponential with an inflection when the tumor volume reaches a threshold where there is a balance between cell proliferation and spontaneous cell death. However, we assume that when tumor cells propagate in a patient, the diffusion volume is considerable, enabling the metastatic disease to expand almost without limit. In addition, when tumors develop resistance to first-line treatments, they usually acquire biological mechanisms favouring accelerated tumor growth. PROM2 could be one of the biomarkers responsible for this runaway metastatic growth. In our research team, we have shown that PROM2 is a predictive biomarker of distant metastases and shorter survival among patients with stage III melanomas [3]. More recently, in a large preclinical study using cancer cell lines and various mouse models of human melanomas, we also demonstrated that the runaway metastatic process is closely linked to PROM2 overexpression, through the increase of epithelial-to-mesenchymal transition (EMT) marker expression and ferroptosis resistance [4]. We report two critical findings: (i) these findings, initially observed in melanoma, have also been confirmed in renal and breast cancers; (ii) we successfully implemented an original *in vivo* model of metastatic runaway in order to mimic what occurs in patients. In this aggravation loop, cancer cell migration induces ferroptotic stress which in turn increases PROM2 expression, and then EMT activation and ferroptosis resistance, promoting metastatic runaway. What makes PROM2 an interesting marker of metastatic disease is the fact that EMT activation and ferroptosis resistance are two hallmarks common to various cancer types.

How can PROM2 be a transtumoral biomarker of metastatic runaway independent from tumor type? Possibly as a result of physiological interplay with membrane lipids, since PROM2 is a cholesterol-binding protein. It could thus be implicated in the redistribution of membrane lipids to form invadopods during the processes of invasion and migration. Concerning ferroptosis resistance, PROM2 contributes to the formation of multivesicular bodies expelling Fe^2+^ from cancer cells. We have also demonstrated that secreted vesicles containing Fe^2+^ are exosomes, which are usually richer in cholesterol and sphingolipids than parental cells. In addition, ferroptosis resistance seems closely related to the type of oxidized phospholipid present in the plasma membrane. In particular, monounsaturated fatty acids (MUFAs) and their ACSL3-dependent acetylation decrease the membrane pool of protective polyunsaturated fatty acids (PUFAs) and induce resistance to ferroptosis [5]. Increased lipogenesis of MUFAs such as palmitoelic acid, with fatty acid synthase upregulation has recently been evidenced as characteristic of cancer pathogenesis. We believe that the role of PROM2 is not restricted to Fe^2+^ expulsion by the cancer cell, but that exosome-expressing PROM2 could prepare the pre-metastatic niche by way of cell-cell signalling and promotion of metastatic spread. Indeed, nanometric extracellular vesicles, including exosomes, play an essential role in the intercellular communication process through their lipid, protein or nucleic acid cargo [6]. Further studies including the multifunctional characterization of exosomes PROM2+ cargo would be of great interest to confirm our hypotheses.

All this makes PROM2 a promising therapeutic biotarget to counteract runaway metastatic disease in different cancer types.
